# Patient decision support resources inform decisions about cancer susceptibility genetic testing and risk management: a systematic review of patient impact and experience

**DOI:** 10.3389/frhs.2023.1092816

**Published:** 2023-05-31

**Authors:** Kelly Kohut, Kate Morton, Lesley Turner, Jonathan Shepherd, Vicky Fenerty, Lois Woods, Chloe Grimmett, Diana M. Eccles, Claire Foster

**Affiliations:** ^1^ Centre for Psychosocial Research in Cancer: CentRIC, School of Health Sciences, University of Southampton, Southampton, United Kingdom; ^2^ Clinical Genetics, St George's University Hospitals NHS Foundation Trust, London, United Kingdom; ^3^ Southampton Health Technology Assessments Centre, University of Southampton, Southampton, United Kingdom; ^4^ Engagement Services, University of Southampton Library, University of Southampton, Southampton, United Kingdom; ^5^ Faculty of Medicine, University of Southampton, Southampton, United Kingdom

**Keywords:** cancer genetics, genetic counselling, patient decision aid, shared decision-making, decision support

## Abstract

**Background:**

Patients with genetic cancer susceptibility are presented with complex management options involving difficult decisions, for example about genetic testing, treatment, screening and risk-reducing surgery/medications. This review sought to explore the experience of patients using decision support resources in this context, and the impact on decision-making outcomes.

**Methods:**

Systematic review of quantitative, qualitative and mixed-methods studies involving adults with or without cancer who used a decision support resource pre- or post-genetic test for any cancer susceptibility. To gather a broad view of existing resources and gaps for development, digital or paper-based patient resources were included and not limited to decision aids. Narrative synthesis was used to summarise patient impact and experience.

**Results:**

Thirty-six publications describing 27 resources were included. Heterogeneity of resources and outcome measurements highlighted the multiple modes of resource delivery and personal tailoring acceptable to and valued by patients. Impact on cognitive, emotional, and behavioural outcomes was mixed, but mainly positive. Findings suggested clear potential for quality patient-facing resources to be acceptable and useful.

**Conclusions:**

Decision support resources about genetic cancer susceptibility are likely useful to support decision-making, but should be co-designed with patients according to evidence-based frameworks. More research is needed to study impact and outcomes, particularly in terms of longer term follow-up to identify whether patients follow through on decisions and whether any increased distress is transient. Innovative, streamlined resources are needed to scale up delivery of genetic cancer susceptibility testing for patients with cancer in mainstream oncology clinics. Tailored patient-facing decision aids should also be made available to patients identified as carriers of a pathogenic gene variant that increases future cancer risks, to complement traditional genetic counselling.

**Systematic Review Registration:**

https://www.crd.york.ac.uk/prospero/display_record.php?ID=CRD42020220460, identifier: CRD42020220460.

## 1. Introduction

Patients who have cancer susceptibility gene testing and are found to carry a pathogenic variant that causes increased future cancer risks (“carriers”) are presented with choices about primary prevention (e.g., risk-reducing surgery, chemoprevention, changes in diet), screening (e.g., earlier, more frequent mammogram/colonoscopy) and treatment for cancer or premalignant conditions (1, 2). They are also encouraged to communicate with at-risk relatives so they can be offered cascade predictive testing (3). Genetic testing has traditionally been supported through genetic counselling: the “process of helping people understand and adapt to the medical, psychological and familial implications of genetic contributions to disease” (4). Genetic counselling promotes personalised, values-based decision-making made possible through formation of a therapeutic alliance during the consultation (5). Increased importance of genomic test results to guide cancer treatment (6–10) and calls for population screening to identity carriers and offer targeted treatment, prevention and surveillance options to high risk groups (11–13) have created increased pressure on already stretched genetics and oncology services. Knowledge can be increased through genetic counselling (14), but people may not accept that risks apply to them (15, 16) or events will happen to them personally, for example due to framing (17) and anchoring-and-adjustment biases (18). Therefore, communicating risks in a personally meaningful way is crucial to quality decision-making.

Shared decision-making between healthcare providers and patients is recommended (19, 20), particularly where choices are personal and complex, as is typical regarding cancer susceptibility genes. Patient decision support resources have been employed in many areas of medicine, including genetics, to promote shared decision-making, streamline clinical consultations and improve decision quality. Patient decision aids (ptDA) are a type of decision support resource that encourage patients to become engaged in difficult decisions by considering not only information about the options, pros and cons but also how personal values influence their decision (21, 22). This is often achieved through the inclusion of a values-based exercise, for example a sliding scale or worksheet for patients to record the importance of personal values relevant to the particular decision. PtDA are useful when there is no clearly preferred option, or when feelings and choices may differ according to individual values. A Cochrane review of 105 studies including 31,043 patients using ptDA before/during clinic compared to usual care revealed increased knowledge and confidence about decisions aligned with personal values, without harmful effects (23). However, clinicians must be mindful that the design and effectiveness of ptDAs is variable, with many not meeting the International Patient Decision Aids Standards (IPDAS) (24) or lacking a theoretical framework (25) to inform content delivery. Also, whilst web-based education may be highly acceptable (26), patients will often seek online sources of information themselves (27), value individual choice, and may not view information if asked to do so at home rather than in clinic (28).

There are two contexts in which patient resources could be relevant to support decisions regarding genetic cancer susceptibility:

i) pre-genetic testing (29): to support patients making a decision about whether to have a genetic test, either with or without a diagnosis of cancer (30).
ii) post-genetic testing: to complement shared decision-making with a healthcare professional for patients identified to have a genetic cancer susceptibility, and their relatives (31, 32).
  At a time of increasing demand for genetic testing and limited in-person resources to support patients, there is a need to better understand the impact and experience of PtDA for genetic cancer susceptibility in both contexts.

This systematic review aimed to identify patient resources to support decision-making pre- testing for any genetic cancer susceptibility, or regarding cancer management options for carriers. The net was cast widely to find any existing resources, including brief educational materials or interventions as well as ptDA, to explore their potential for supporting patients. A secondary goal was to highlight gaps to guide future work co-designing a PtDA with patients.

The extent to which existing decision support resources meet patients' needs and preferences was explored in terms of:

i) impact on outcomes, e.g. cognitive, emotional, or behavioural.
ii) patient experience.

Recommendations for clinical practice and future research were proposed.

## 2. Methods

The Centre for Reviews and Dissemination's guidance for reviews in health care (33) and the Preferred Reporting Items for Systematic Reviews (PRISMA) statement (34) guided methods and reporting for this systematic review. The protocol was published on PROSPERO: https://www.crd.york.ac.uk/prospero/display_record.php?RecordID = 220460.

### 2.1. Co- design

Stakeholders were consulted from the earliest planning phases and throughout this review, includingpatient engagement in design and data synthesis. Patients have been included from the Cancer Research UK-funded CanGene-CanVar programme. An International Lynch Decision Aid Stakeholder Panel has been established to provide advice and guidance. Individuals have been invited based on expertise in clinical care, academic work, public policy, patient charities, peer support groups and public bodies.

### 2.2. Literature searching

The following databases (and host platforms) were searched and re-run prior to final analysis (from database inception to 02/07/2021, English language only): MEDLINE (EBSCOhost), PsycINFO (EBSCOhost), Embase (OVID), CINAHL (EBSCOhost), Web of Science Core Collection, and the Cochrane Library (Cochrane Database of Systematic Reviews (CDSR); Cochrane Central Register of Controlled Trials (CENTRAL)). The search strategy combined key word and subject terms targeting three concepts: cancer genetics, decision-making, and written resources (Supplementary Material Table S1).

In addition, other relevant studies were identified by examining bibliographies of included publications and using forward citation searching in Web of Science Core Collection. Grey literature was searched to identify unpublished resources (Supplementary Material Table S2).

### 2.3. Study selection

Inclusion and exclusion criteria are detailed in Table 1. In brief, studies were included if they involved adults (with or without cancer) who used a decision support resource pre- or post- testing for any cancer susceptibility genes. These could be delivered digitally or paper-based, and included one or more of the following: information, education, visual presentation of cancer risk and personalised resources, including ptDA. Quantitative, qualitative and mixed methods studies were included.

**Table 1:** Study inclusion and exclusion criteria.

**Population**	**1. People with a cancer diagnosis deciding about:** a) genetic testing b) treatment or risk-reducing options post-genetic testing **2. People with a known pathogenic variant in a cancer predisposition gene deciding about risk-reduction options3. People at increased risk deciding about genetic testing, including:** a) people with a family history of cancer b) people with a personal history of cancer c) people with a known pathogenic variant in the family deciding about predictive testing d) people of Ashkenazi Jewish descent **Exclusion:** 1. Under 18 years of age 2. Parents making decisions on behalf of their children 3. General population without any known raised cancer risk or with a family history using a resource or consultation to consider whether they are eligible for referral for genetic testing 4. People not at raised risk asked to consider hypothetical risk
**Intervention**	Written or pre-recorded patient-facing resources, including information, education, risk presentation, and decision support. Digital (e.g. web-based, email, smartphone, text messaging, non-live webinars) or paper-based. **Exclusion:** 1. Genetic counselling sessions without giving patients a digital/written resource 2. Risk prediction models at population level to inform HCPs or guidelines 3. Resources to help HCPs identify patients for referral to genetic testing, e.g. family history questionnaire 4. Social media and patient fora 5. Resources to support people to cope with the process of genetic testing 6. Resources to facilitate communication with family members 7. Resources to facilitate reproductive decisions 8. Resources not available in English
**Comparator**	Control group if the study has one, but not necessary
**Outcomes**	Quantitative or qualitative evaluations of acceptability and impact of decision support resource, including cognitive outcomes (e.g. knowledge, intention to use genetic testing, perceived risk), emotional outcomes (e.g. satisfaction, decisional conflict, emotional burden, anxiety) and behaviour change following test results. Studies describing resource development process only included if impact or experience of patient captured in some way. **Exclusion:** 1. Studies which examine factors influencing decision-making, but which are not focused on the impact of a written resource on this process. 2. Studies which do not report any patient outcomes of interest to the review.
**Study design**	Any

All search results were exported to EndNote X9 software for de-duplication. Two reviewers (KK, KM) independently screened 20% of titles and abstracts (sampled in alphabetical order) as a pilot to test whether the inclusion/exclusion criteria were appropriate and to assess whether their application was accurately applied by both reviewers. Rayyan, a web application for collaboration on systematic reviews (35) was used. After each batch of 100 references in the 20% pilot, reviewers' decisions were unblinded and compared. There were 29 disagreements out of 500 and these were all resolved through discussion. Informed by the pilot, the eligibility criteria were adjusted following discussion with the wider research team (DE, CF, CG, LT). The remaining 80% of titles and abstracts were screened by the lead author (KK). Both reviewers completed full text screening. Where discrepancies arose, discussion took place (involving the wider research team where necessary) until agreement was reached.

### 2.4. Data extraction and critical appraisal

Data from eligible publications were extracted into an Excel database with fields guided by the TIDieR (template for intervention description and replication) checklist (36). Both reviewers performed independent data extraction for 10% of the included studies. Disagreements were resolved through discussion. Subsequently, KK extracted data from the remaining studies, checked by the second reviewer.

Critical appraisal was performed by KK and KM (Supplementary Material Table S3). Depending upon the type of study, this was guided by National Institute for Health and Care Excellence (NICE) checklists for quantitative intervention studies and qualitative studies (37) or the mixed-methods appraisal tool (38). Aspects of study design and reporting were appraised. Each study was awarded an overall study quality grading for internal validity and external validity and subject to an overall assessment grading of how well the study was conducted, as far as could be ascertained from the paper. Appraisal of study criteria in Supplementary Material Table S3 is presented as yes, no, or N/A (not applicable or not able to ascertain from the paper).

### 2.5. Data synthesis

**Figure 1:**
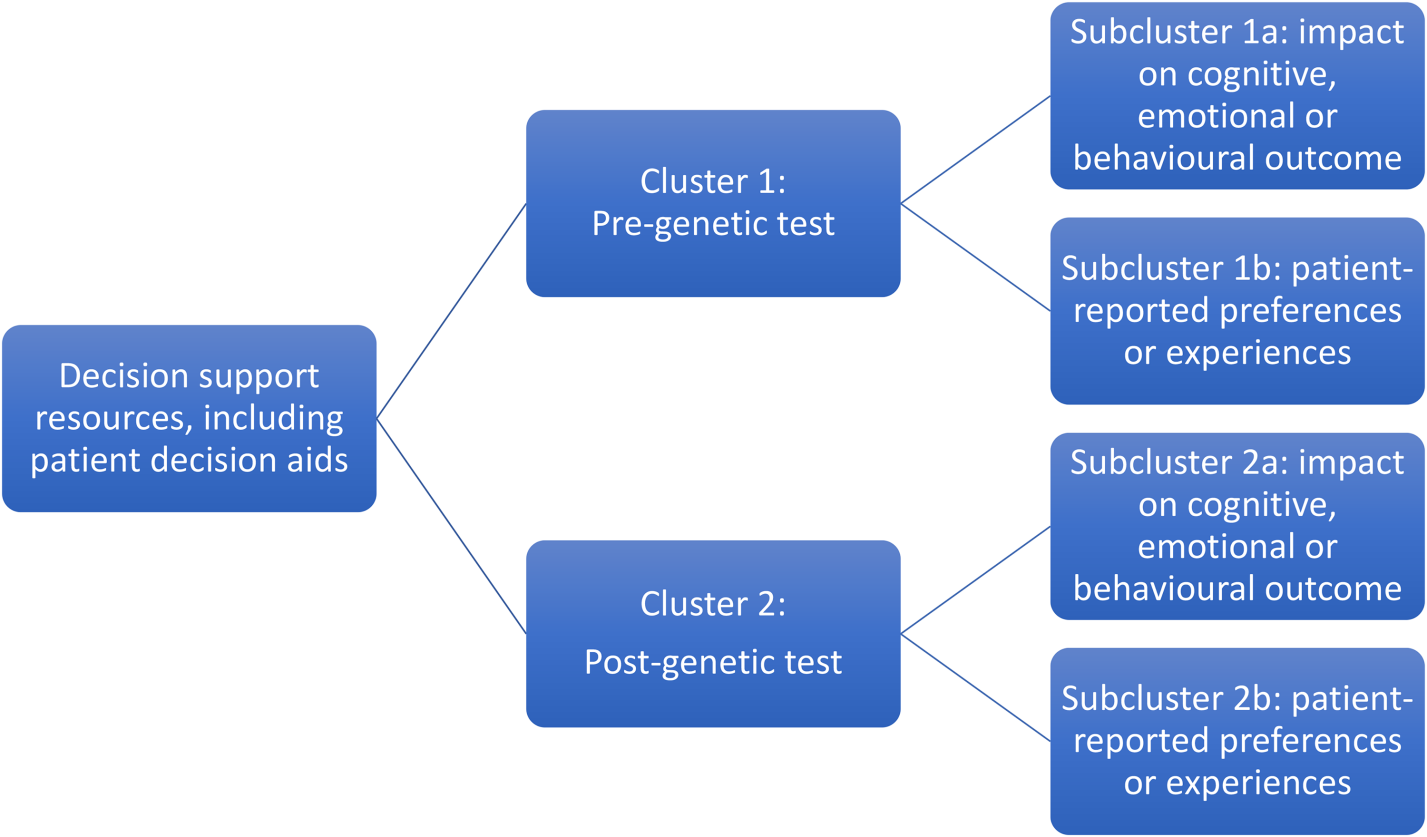
Studies included in the systematic review were grouped into clusters based on the context of the patient decision support resource (pre- and post-genetic testing) and subclusters relating to the impact on outcomes and patient-reported experiences.

The review aims necessitated inclusion of a wide range of studies, mostly of quantitative but also mixed methods and qualitative design. Meta-analysis was not possible due to the heterogeneity of methodologies, populations and outcome measures. Narrative synthesis, often used for systematic reviews of healthcare interventions (39), was selected as the most appropriate method to synthesise findings without diluting the individual value contributed by different study designs (40, 41). Tabulated data were examined to describe patterns and summarise estimates of effect direction and size (39, 42). Studies were grouped into clusters and subclusters based on pre- or post-genetic test setting and patient outcomes (Figure 1) to facilitate post-hoc subgroup analysis (40). Qualitative data were subjected to thematic analysis to interpret primary themes and concepts, and representative patient narratives were chosen to be presented in their original form to highlight these themes (41).

## 3. Results

**Figure 2:**
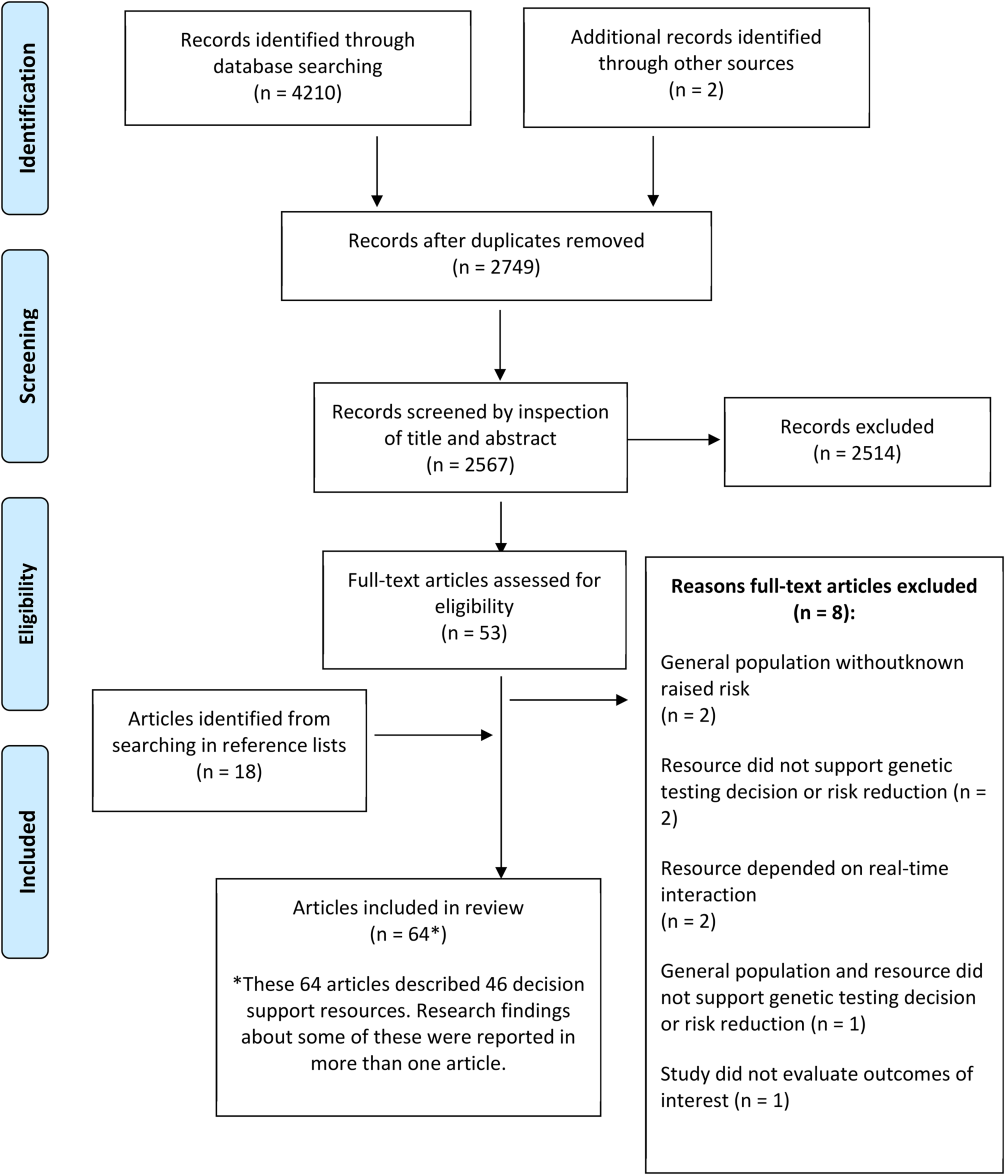
Flowchart showing the flow of study selection for the systematic review.

**Figure 3:**
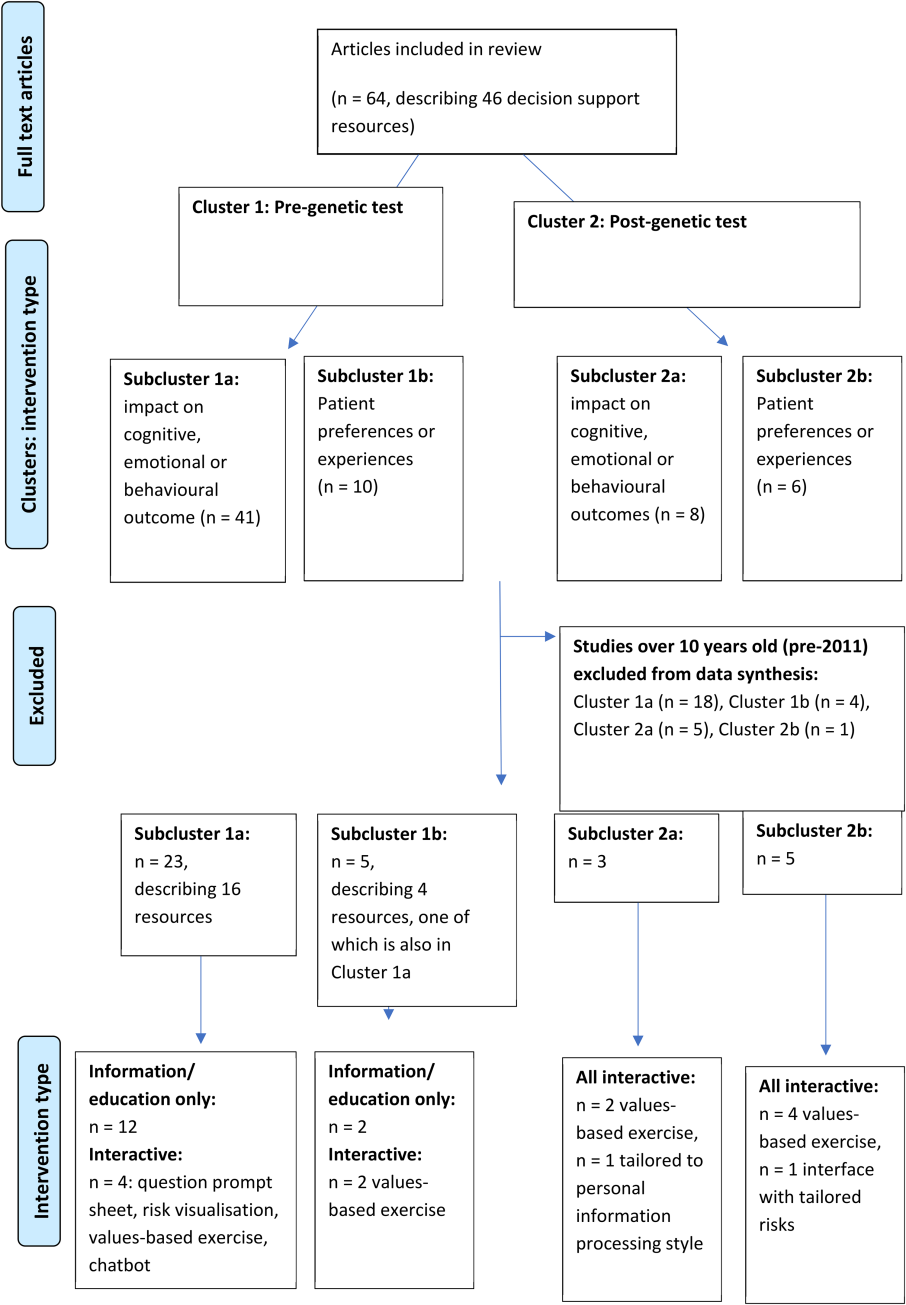
Studies included in data synthesis, grouped into clusters and subclusters.

Sixty-four publications regarding 46 patient decision support resources were found to be eligible from the searches (more than one publication was included regarding some of these). Figure 2 shows the flow of study selection using the Preferred Reporting Items for Systematic Reviews and Meta-Analyses (PRISMA) flow diagram (34). Most studies were from United States (*n* = 26), followed by Australia (*n* = 12) and Netherlands (*n* = 12). A pragmatic decision was taken to focus data synthesis on studies published from 2011 onwards, and the protocol was amended accordingly. Older studies were based on outdated guidelines and less likely to be relevant to our study aim of identifying resources that could be used or easily adapted for current practice. As shown in Figure 3, 36 publications were retained, describing 27 resources divided into two clusters (pre-and post-genetic test) and four subclusters (impact on cognitive, emotional or behavioural outcomes and patient-reported preferences/experience for each cluster) as illustrated in Figure 1.

### 3.1. Critical appraisal of studies

Most (*n* = 17) studies contained strong certainty of evidence/low risk of bias, 13 contained medium certainty of evidence/moderate risk of bias and three contained weak certainty of evidence/high risk of bias (Supplementary Appendix S3). Study design varied, from publications describing development of resources with some preliminary patient evaluation in a hypothetical decision-making setting (32, 43–50), to larger randomised controlled trials in target populations (51–56). Follow-up was often short (less than two months), with only a few studies recording outcomes as long as 12 months after resource use (53, 55, 57–59). Qualitative study designs resulted in some rich findings (60–64) but some lacked rigorous analysis methods leading to more shallow data (45, 60). Overall, external validity was limited by lack of patient diversity.

### 3.2. Target population

Cluster 1 resources supported pre-genetic test decisions for patients diagnosed with breast (28, 57, 60, 61, 64, 65, 66, 67), ovarian (68), breast/ovarian (69) and colorectal cancer (70, 71) as well as patients (mostly) unaffected by cancer with a family history (48, 49, 53, 54, 72, 73) or of Ashkenazi Jewish heritage (51, 62) (see Table 1). All Cluster 2 resources targeted *BRCA1* or *BRCA2* carriers to support decisions about risk management post-genetic test. These were designed for carriers unaffected by cancer (45, 55), with personal history of breast cancer (63), separate versions tailored to breast cancer history (32) or not specified (47, 46, 52, 59).

#### 3.2.1. Setting for delivery

Cluster 1 resources were designed to replace face-to-face genetic counselling pre-test (28, 48, 57, 62, 67, 68, 69) or to supplement genetic counselling (44, 49, 51, 53, 54, 60, 64, 66, 70, 72, 73), which may have been delivered in the mainstream setting by oncology professionals (52, 65, 71). Cluster 2 resources were exclusively to supplement standard of care genetic counselling post-test for carriers.

### 3.3. Characteristics of decision support resources

#### 3.3.1. Conceptual/theoretical framework

In most publications, a conceptual/theoretical framework to inform design was not specified. However, six resources were based on underlying theory (43, 49, 53, 54, 59, 64, 66, 71, 74) (see Table 2). These included theories related to information tailoring (43, 53, 74), such as the elaboration likelihood model of communication persuasion, which examines how presenting personalised messages can encourage more thoughtful decision-making (75, 76). The health belief model (77) suggests behaviour change is maximised if resources address threat severity and personal benefits and the transtheoretical model of health behaviour change (78) describes stages of change that patients move through before taking action. These two behaviour change theories informed content in psychoeducational resources and measured intention to pursue genetic counselling and testing (66). Another theory informed information presentation (64, 66): the fuzzy-trace model postulates decision-making is influenced by a quick, intuitive “getting the gist” which is personal and values-based, and can be more important than memory of information learned verbatim (76).

**Table 2:** Characteristics and main findings of studies.

Authors (year), country, time of recruitment	Study type	Description of resource/intervention	Theoretical underpinning/framework	Population	Setting	Intervention: number of subjects	Comparator	Control: number of subjects	Main outcomes	Main findings
**Cluster 1a. Deciding whether to have a genetic test: intervention impact on cognitive, emotional or behavioural outcome (*n* = 22 studies, describing 15 interventions)**										
Albada et al. (2011), Netherlands, 02/2008–04/2010	Process evaluation (quantitative)	E-info gene^ca^: patient-facing website about breast cancer genetic counselling tailored on age, cancer history, extensiveness of information, with question prompt sheet (QPS)	Elaboration likelihood model	Consecutive female counsellees aged >18 years, no familial pathogenic variant	At home, prior to pre-test genetic counselling	101, of which 85 (84.2%) completed evaluation	NA	NA	factors influencing use, evaluation of website	All counselees used website, mean time 21 min (range 39s-1 h36 m), with those affected by breast cancer spending longer. Most selected extensive detail, but 8/85 (9.6%) then found information too much and 12 (14.4%) upsetting. Counselees highly satisfied with website. Those with greater information needs found most helpful. Less than half viewed genetic counselling information. Forty-two (41.7%) formulated questions to discuss at appointment.
Albada et al. (2012)	RCT					101	brief leaflet before pre-test genetic counselling	89	genetic counselling expectations, knowledge, information needs	Use of tailored website prior to genetic counselling resulted in more realistic expectations (*p* = 0.03) and reduced requirement for education, freeing up time for Genetic Counsellor to focus on personalised risk communication and psychosocial factors.
Albada et al. (2015)	RCT					86		76	genetic counselling satisfaction, knowledge, anxiety, risk perception, surveillance adherence	One-year follow-up revealed higher satisfaction (*p* = 0.02) and more perceived personal control (*p* = 0.02) in intervention group. However, overestimation of cancer risk persisted and no significant improvement in knowledge, anxiety or adherence to recommendations.
Arrick et al. (2019), USA, dates not specified	RCT	Visual aids with calculated vs self-perceived 10-year cancer risk: 1) spinner pie chart game, 2) computer-generated random dot arrays	not stated	consecutive counselees	During clinic, to supplement pre-test genetic counselling	n/66	pre-test genetic counselling with 10-year cancer risk calculation	n/66	accuracy of risk perception	More than 85% of counsellees overestimated cancer risk by at least two-fold before genetic counselling. Enhanced counselling significantly improved risk perception accuracy, sustained at two-week and six-month follow-up for both spinner game “out of 1,000” and random dot array.
Cragun et al. (2020), USA, 11/2018–03/2020	Process evaluation (quantitative)	Educational tool: twelve-minute narrated, animated slides about multigene panel testing	Not stated	Consecutive counsellees aged ≥18 years	Via tablet at hospital before pre-test genetic counselling	305	NA	NA	knowledge, empowerment, attitudinal values about genetic testing, evaluation of health literacy relationship with outcomes	Intervention significantly increased knowledge, based on unvalidated questionnaire. Genetic testing decision empowerment increased (29% to 74%, *p* < 0.001), but was lower before-and after-tool with lower health literacy. Values about genetic counselling did not change. Triage of counsellees to identify lower health literacy and/or sustained decisional conflict could identify need for traditional pre-test genetic counselling.
Dekker et al. (2014), Netherlands, CRC < 70 01/-07/2009, all CRC 06/2010–01/2011	RCT	Novel strategy (NS): patient/clinician website about familial CRC risk, with risk calculators and decision support intervention (DSI) for high risk patients, patient brochure	Not stated	Patients newly diagnosed with CRC, no known pathogenic variant	At home, prior to usual care	140	usual care	252	cancer prevention measures (genetic counselling for high risk, colonoscopy in first degree relatives for moderate risk)	Website used by 94/140 (67%). Genetic counselling uptake lower (15% vs 33%, *p* = 0.003) in high risk after NS. No significant difference in cancer prevention measures in moderate/low risk, but familial risk discussed more frequently. Although web- and paper-based tools appreciated as supplement to improve knowledge, communication and shared decision-making, CRC patients preferred doctor's advice when considering cancer prevention.
Gornick et al. (2018), USA, 02/2014–05/2016	RCT (secondary analysis of data collected in RCT)	iCan-Decide: web-based interactive decision tool with values-based exercise and testimonials tailored to age, ethnicity and timing of breast surgical consult	IPDAS	Patients newly diagnosed early stage breast cancer aged 21–84 years enrolled at surgical practice	At home or on tablet at hospital; prior to or after surgical consultation	245	static (non-tailored) website	251	knowledge, uptake of genetic testing	Knowledge regarding probability of BRCA1 or BRCA2 pathogenic variant low but significantly increased (35.8% vs 24.4%, *p* < 0.006) after intervention. However, knowledge also related to educational status, younger age and ethnicity, suggesting potential for more impact in subgroups. Website complements but should not replace professional advice and need to address HCP knowledge gaps.
Hall et al. (2011), USA, 06/2005–12/2008	RCT	CD-ROM about MSI testing: graphics and pictures with professional narration, based on structural equation modelling	Theoretical model of association between knowledge, attitudes and decisional conflict	Patients diagnosed CRC offered MSI testing, did not meet Amsterdam criteria	At hospital, after education session	120	brief description of MSI and IHC by health educator	119	knowledge, self-efficacy, preparedness, decisional conflict	Decision aid not tailored, did not address barriers. Knowledge regarding MSI increased in intervention group (*p* < 0.05) along with preparedness for decision-making (*p* < 0.05). Decisional conflict mediated through multiple pathways including knowledge-independent and attitude.
Heald et al. (2020), USA, 05/2019–03/2020	Process evaluation (quantitative)	Chatbot-deployed education and consent for 55 pan-cancer gene panel	Not stated	Patients presenting for colonoscopy aged ≥18 years	At home, *via* electronic medical record patient portal	487	NA	NA	feasibility, uptake of genetic testing	Chat initiated by 487/4,254 (11.4%). Genetic testing ordered for 161 (chat) + 8 (*via* Genetic Counsellor). No Lynch Syndrome identified. Twelve (9.3%) had pathogenic variant identified, four with known personal/family history of hereditary syndrome. Chatbot was used as a genetic counselling extender, evaluating three times as many patients compared to traditional counselling time in previous study.
Hoberg-Vetti et al. (2016), Norway, 09/2012–04/2015	Cohort analytic (two groups pre- + post-)	DNA-BONus study: written information on hereditary breast and ovarian cancer, testing BRCA1 and BRCA2 Norweigian founder pathogenic variants	Not stated	Patients newly diagnosed breast/ovarian cancer recruited from surgical/gynaecological clinics	In oncology clinic, in place of pre-test genetic counselling	1,015 (breast cancer *n* = 405, ovarian cancer *n* = 83)	NA	NA	uptake of genetic testing, anxiety, depression	Genetic testing accepted by 68.0% with ovarian and 45.4% with breast cancer. Older patients may have declined study. Baseline anxiety/depression similar to newly diagnosed patients with cancer in general. Anxiety significantly decreased by six-months (*p* < 0.001), during which time genetic testing was completed. No change in depression. Only 20 patients called Genetic Counsellor with questions.
Hoberg-Vetti et al. (2019)	Cohort analytic (two groups pre- + post-)					309 (breast cancer *n* = 259, ovarian cancer *n* = 50)	NA	NA	cancer-related distress, perceived social support, decisional conflict	Participants psychological substudy (309/1,015, 40.0%) significantly younger. Severe stress response decreased from baseline to six-months’ post-test for intrusion (32.1 to 14.0%) and avoidance (23.6 to 16.0%). Factors for increased cancer-related distress included younger age, ovarian cancer, less perceived social support, higher decisional conflict.
Kasting et al. (2019), USA, 03/2015–09/2015	RCT	Psychoeducational intervention (PEI): booklet, 12-minute DVD about hereditary breast cancer featuring patients, oncologists, genetic counsellor	Health Belief Model, Transtheoretical Model of Behaviour	Patients post-surgery for breast cancer, ‘high risk’ due to diagnosis aged ≤50 years or family history	At home, prior to pre-test genetic counselling	53	one-page factsheet: information about hereditary cancer and appointment logistics	56	feasibility, acceptability, preliminary efficacy, cancer worry and distress	Based on self-report 88% viewed video. Intervention group greater intention to pursue genetic counselling after four-months (28.0 vs 7.7%, *p* = 0.027), although only 3% had attended. PEI no effect on perceived barriers or cancer worry, both factors for behaviour change. Control group greater decrease in perceived cancer risk (*p* = 0.064).
Manchanda et al. (2016), UK, 02/2009–07/2010	RCT	GCaPPS study: DVD presentation to small groups (2–5) about testing for BRCA1 and BRCA2 Ashkenazi Jewish founder pathogenic variants	Not stated	Ashkenazi Jewish aged >18 years, no known familial pathogenic variant, recruited from community	during clinic, followed by individual pre-test genetic counselling	409	pre-test genetic counselling	527	knowledge, genetic testing uptake, perceived cancer risk, counselling time/satisfaction	DVD non-inferior with respect to knowledge, counselling satisfaction and perceived risk, and decreased appointment time by mean 20.5 min (*p* < 0.005), saving £14 per person. DVD well received: 98% satisfied with length/information, 87–95% not significantly worried, 89% proceeded with genetic testing.
McCuaig et al. (2019), Canada, 05/2015–03/2018	RCT	Prevent Ovarian Cancer Program: 20-minute, voice-recorded presentation about 52-gene panel testing for hereditary ovarian cancer	Tiered and binned approach to informed consent for multigene panel testing (Bradbury et al. 2015)	Females aged ≥18 years, FDR died ovarian cancer, no known familial pathogenic variant, recruited from community	At home, prior to brief, telephone pre-test genetic counselling	237	pre-test genetic counselling (option *via* video if not local)	116	knowledge, genetic testing uptake, perceived cancer risk, distress, anxiety, depression, decisional conflict, counselling time/satisfaction	Intervention non-inferior for knowledge, distress, anxiety, depression, decisional conflict but not perceived cancer risk. There was a 26-minute time savings (*p* < 0.001). Nearly all (>90%) participants in both groups pursued genetic testing, 86%–90% wanted all gene results. Both groups highly satisfied.
Meiser et al. (2012), Australia, dates not specified	Process evaluation (qualitative)	One-page educational pamphlet with information about treatment-focused BRCA1 and BRCA2 genetic testing (TFGT)	Not stated	Females aged ≥18 years with breast cancer diagnosed aged ≤50 years	At home, retrospective (previously tested) or hypothetical (recently diagnosed)	17	NA	NA	acceptability, preferred timing and impact of pamphlet	Semi-structured interviews that informed pamphlet development suggested patients preferred to be told about TFGT at or around time of diagnosis in face-to-face consultation with HCP. Sixteen out of 17 patients satisfied with information provided, 15 self-reported improved understanding, and only four were worried by reading the pamphlet.
Quinn et al. (2017), Australia, 07/2010–10/2012	RCT	see Meiser et al. 2012		Females aged 18–49 years with breast cancer, no prior genetic testing, high risk	At home, in place of pre-test genetic counselling with brief Genetic Counsellor telephone intake	65	traditional pre-test genetic counselling	70	decisional conflict, cost effectiveness	All 135 patients consented to testing, 20 (14.8%) had a pathogenic variant and 18 (13.3%) had a variant of uncertain significance. Pamphlet saved AUD$84 per patient and not inferior with respect to decisional regret at 12-months’ follow-up for genetic testing or risk-reducing surgeries.
Nilsson et al. (2018), Sweden, 02/2015–08/2016	Process evaluation (quantitative)	BRCAsearch study: short letter about BRCA2 and BRCA2 testing with invitation to contact Genetic Counsellor if required	Not stated	Patients with newly diagnosed breast cancer taking part in Swedish Cancerome Analysis Network biobank study	Oncology clinic, in place of pre-test genetic counselling	818	NA	NA	uptake of genetic testing, number of patients contacting genetic counsellor	Of 818 patients given the letter, 542 (66.2%) consented to genetic testing. Eleven pathogenic variant carriers were identified (2.0% prevalence), six of whom fulfilled Swedish testing criteria. Only 11 (2.0%) contacted Genetic Counsellor with questions, and 19 (3.5%) had questions regarding administrative processes.
Nilsson et al. (2019)	Process evaluation (quantitative)					805	NA	NA	patient-, tumour- and treatment-related predictors of genetic testing uptake	Analysis from medical records in 539/805 (67%) who consented to genetic testing. Consenting patients younger (mean 61.9 vs 67.1 years, *p* < 0.001), more educated (*p* = 0.003) and more likely to have family history (*p* = 0.02). Patients with psychiatric disorders less likely to have testing (*p* = 0.006).
Nilsson et al. (2019a)	Process evaluation (quantitative)					448	NA	NA	one-year post-results satisfaction	Satisfaction 96.0%; only 11.1% preferred more oral information, more likely with comorbidities (*p* = 0.02), born outside Sweden (*p* = 0.01), lower education (*p* = 0.06). Twenty-nine contacted genetic counsellor and had pre-test counselling. All 11 pathogenic variant carriers satisfied with testing decision, 10/11 with information; all attended post-test counselling and had risk-reducing BSO.
Sie et al. (2014), Netherlands, 08/2011–02/2012	Cohort analytic (two groups pre- + post-)	DNA-direct study: triage call by doctor followed by written and digital information (website, educational movie) about BRCA and BRCA2 testing.	Not stated	Females (previously) diagnosed breast cancer, referred to Genetics, no known familial pathogenic variant	At home, in place of pre-test genetic counselling	95	DNA intake: pre-test genetic counselling	66	choice of DNA-direct vs. DNA-intake, satisfaction with genetic counselling, distress	More patients (59%) chose streamlined DNA-direct (*p* = 0.03), received results quicker (70 vs 103 days, *p* = 0.002), and 89% would choose again. DNA-direct more informed, lower decisional conflict, and lower distress baseline and two-week follow-up (*p* = 0.001). Identification of pathogenic carriers 8% in both groups. Satisfaction with genetic counselling equal. Most DNA-direct did not view video, only read letter, and none contacted doctor with clinical questions.
Sie et al. (2016)	Cohort analytic (two groups pre- + post-)					59		49	one-year post-results satisfaction, distress, Genetic Counsellor experience	Both self-selected groups satisfied with choice of procedure, with no regret at one-year. DNA-direct lower distress, although both groups below clinical thresholds and difference smaller at one-year. Genetic Counsellors who performed post-test counselling believed most (76%) DNA-direct patients made informed choice for genetic testing and felt 85% were suited for streamlined model.
Tea et al. (2018), Austria, 02/2015–02/2016	Process evaluation (quantitative)	New Genetic Counselling Tool (NGCT): visual aid about BRCA1 and BRCA2 testing using lay language, with pictures, diagrams and tables	Not stated	Patients aged >18 years referred for genetic counselling	During clinic, to supplement pre-test genetic counselling	34	pre-test genetic counselling (without visual aid)	36	knowledge	More NGCT (44% vs 17%, *p* = 0.012) correctly answered all seven knowledge questions correctly on unvalidated post-counselling questionnaire. Visual aid was used in genetic counselling sessions, and effect on counselling style and language not measured in this study.
Watson et al. (2016), USA, 7/2014–12/2014 (standard), 3/2015–8/2015 (video)	Process evaluation (quantitative)	Seven-minute educational video explaining choice between BRCA1 and BRCA2 testing with reflex to multigene panel	Not stated	Patients with ovarian, fallopian tube or primary peritoneal cancer	During oncology clinic, on a tablet, in place of pre-test genetic counselling	295	pre-test genetic counselling	267	uptake of genetic testing	Fifty-five per cent (162/295) of patients who viewed video consented to testing on same day, a significant increase (*p* ≤ 0.001) compared to previous referral method. Detection of pathogenic variants similar in both groups (8.1 vs 7.9%).

Whilst not all ptDA were informed by theory, some (32, 45, 49, 55, 59, 63, 64) followed recommended guidelines such as the Ottawa Decision-Support Framework (ODSF) (79) which includes a values-based exercise to improve decision quality and process. Similarly, the multiattribute value and utility models (80, 81) guided inclusion of pros and cons of cancer risk management choices that patients rated according to personal importance (59), and the informed choice model (82, 83) was used to theorise that increased knowledge would improve decision-making quality and genetic testing uptake (49).

#### 3.3.2. Format and content delivery mode varied widely

For studies aimed at replacing the need for in-person counselling pre-test, a brief information letter was used (57, 69, 67), or letter and digital options including a website (49), video (28) or chatbot (48). Educational presentations to supplement genetic counselling ranged in length from seven-minute video (68), 12 minute DVD (66) or animated slides (73), 15 minute DVD delivered to small groups (51), 20 minute voice-recorded presentation (54) and CD-ROM about microsatellite instability testing in colorectal cancers that took on average 24 min to view (71).

Paper-based resources were either brief (one-page) and focused on treatment-related implications of genetic testing for people with breast cancer (60) or longer (15-page) including a worksheet to record personal weighing up of options (32, 47, 55). Others made resources available online with printable content (45, 64). Visual aids were created to improve genetic cancer risk communication using pictures, diagrams and tables (44) or by comparing an interactive spinner-game format to random dot icon arrays (72). Digital decision support resources were mostly interactive (62, 63, 64, 52, 59, 70) and some also included computer-tailoring for personal characteristics to present more relevant information (46, 49, 53, 65).

### 3.4. Impact of decision support resources

A summary of the main findings is presented in Table 2, with narrative synthesis below.

#### 3.4.1. Knowledge, understanding and expectations

Improvement in genetic cancer susceptibility knowledge or realistic expectations was statistically significant for many (44, 51, 53, 54, 65, 71) but not all resources (70). However, measurement was often by self-report, using unvalidated questionnaires and/or with lack of pre- and post-counselling measures or control group. In one study, patients stated viewing an educational video increased knowledge, however qualitative data gathered *via* semi-structured interviews suggested gaps remained, for example many thought genetic counselling was the same as genetic testing (61).

Accuracy of cancer risk perception significantly improved by adding personalised visual aids to genetic counselling (72), with effects sustained up to six-months. However, recorded presentations before a shortened session had mixed results when compared to traditional counselling in randomised trials. An educational video followed by abbreviated genetic counselling was non-inferior to traditional counselling for increased knowledge, satisfaction and risk perception for community-recruited participants offered Ashkenazi Jewish founder *BRCA1* and *BRCA2* gene testing (51). Patients with a relative who died of ovarian cancer were offered 52-gene panel testing using a digital presentation viewed at home followed by short telephone genetic counselling, which was non-inferior to traditional counselling for knowledge, satisfaction and psychological factors but not for ovarian cancer risk perception (54).

#### 3.4.2. Distress, anxiety and cancer worry

Mental well-being outcomes such as distress, anxiety, cancer worry and depression tended to be below clinically relevant thresholds at baseline and follow-up, indicating no significant evidence of psychological harm from pre-test resources (51, 53, 54, 66, 67, 69, 84). For people newly diagnosed with cancer, levels were transiently increased as expected at this challenging stage of life;genetic testing using decision support resources did not increase symptoms (28, 58, 66, 67, 69). However, several studies excluded patients with psychological conditions (28, 57, 58, 60, 85, 86), so it is not known how resources might have influenced mental well-being in these groups.

Some post-test resources showed time-dependent results, with cancer-related distress higher in the PtDA group compared to usual care at one month, but lower from one- to six-months, possibly indicative of a deliberative decision-making process (59) or declining over time but similar in ptDA and usual care groups (55). Distress was also shown to vary by topic, with lower levels regarding chemoprevention compared to risk-reducing breast and ovarian surgery decisions (55).

#### 3.4.3. Genetic testing uptake

Written information instead of pre-test counselling led to genetic testing uptake in 405/1015 (45.4%) (84) and 542/818 (66.2%) (85) patients with breast and 83/1,015 (68.0%) with ovarian cancer (84), although there was no comparator group and older people were less likely to take part. An educational video followed by brief counselling in community-recruited patients led to high testing uptake, with 92% of video vs. 96% of the traditional counselling group electing testing, and video delivered a significant time saving (19.4 vs. 45.8 min, *p* < 0.001) (54). In a similar study, 89% across DVD and traditional counselling groups had testing, with the DVD saving 20.5 min of counselling time (51). However, there was lower uptake of testing amongst patients with ovarian cancer shown a video in their oncology clinic instead of referral for genetic counselling (162/295, 55%) (68). Despite interventions increasing knowledge and interest in testing, patients particularly valued their doctor's advice and did not always follow through on scheduling a genetic counselling appointment (65, 66). Some resources were used to manage expectations about being offered testing (48, 56), a successful approach for people at lower risk who do not meet current eligibility guideline criteria.

#### 3.4.4. Decisional conflict

Decisional conflict describes level of uncertainty about making a choice. The decisional conflict scale measures contributing factors such as support, information and personal values (87). Results from this review suggest patients with higher decisional conflict may need additional decision support and genetic counselling (57, 73, 65).

An educational resource to shorten pre-test genetic counselling was non-inferior to standard care with respect to decisional conflict in patients with a family history of ovarian cancer (video (54),) and patients diagnosed with breast cancer before age 50 years (pamphlet (67),). Baseline levels of decisional conflict were moderate in patients referred for genetic risk assessment, and showed significant decrease at two-months after using an interactive, web-based ptDA about breast reconstruction after risk-reducing mastectomies, compared to standard of care counselling (52). One study in 239 high risk patients with colorectal cancer (71) showed that a ptDA impacted decisional conflict by increasing knowledge and preparedness to make a decision. Decisional conflict was also influenced by knowledge-independent factors such as attitudes about testing and learning about hereditary cancer risk which, along with other barriers, are often not addressed in ptDA.

*BRCA1* and *BRCA2* carriers with no history of cancer had low baseline decisional conflict about breast risk management options in intervention and control groups, which declined with time up to 12-months and was not significantly influenced by a paper-based ptDA used at home after post-test genetic counselling (55).

### 3.5. Evaluation of decision support resources

#### 3.5.1. Satisfaction and acceptability by patient report

Satisfaction and acceptance was high across clinical settings and patient groups; however, resource usage was often untracked, and many studies did not compare to standard care in a randomised controlled trial. Where optional usage was monitored or self-reported, this revealed 64/100 (64%) used an interactive CD-ROM (59), 94/140 (67%) used a website (70), and 53/60 (88%) viewed a video (66). A much lower percentage (487/4,254, 11.4%) of patients presenting for colonoscopy screening engaged with a chatbot to answer questions about their family history to determine eligibility for genetic testing (48). Most (95/161, 59%) patients with breast cancer chose to use streamlined pre-test information instead of genetic counselling; when presented with letter and video options, most only read the letter and none contacted the doctor with questions (28). There was no regret at 12-months about choosing streamlined testing, which identified a pathogenic BRCA variant in 8/95 (8%) of patients (58). Similarly, 96% of patients with breast cancer were satisfied at 12-months with a short letter instead of pre-test counselling and only 11/818 (2%) contacted the genetic counsellor for support (57). Only 20/1,015 (1.9%) of patients with breast or ovarian cancer who received brief written pre-test information in oncology contacted the genetic counsellor (84).

#### 3.5.2. Experience and emotional outcomes by patient report

Two studies explored the experience of patients with breast cancer using resources to decide about genetic counselling/testing. In a telephone structured interview study to evaluate acceptability and emotional impact of a one-page pamphlet about treatment-focused genetic testing, 7/17 people thought the pamphlet sufficient for decision making, whilst 10/17 believed more information was needed, e.g., discussion with healthcare professional (HCP) or searching online (60). Four out of 17 were worried by reading the pamphlet: three were reminded of their breast cancer diagnosis and one was worried about their relatives. Think-aloud interviews reviewing a web-based ptDA revealed patients with breast cancer preferred less text, to get the “gist”, with optional, more detailed information, and wanted a “friendlier” feel to patient pictures (64). This is in contrast to study findings about another web-based ptDA tailored for personal characteristics (43), in which patients with breast cancer spent more time looking at information and selected to receive extensive detail, however when looking at the information 12/85 (14.4%) then found it upsetting.

The JeneScreen web-based programme for Ashkenazi Jewish BRCA testing was evaluated in a pre- and post-test interview study of 11 patients without cancer (62). Similar to findings from another study involving patients with breast cancer (64), some wanted less pre-test information up front and suggested a staged approach: “*…But you may not get that result, so you wouldn”t need to go into as much detail about that topic…only if you need the information”.* Ten out of 11 were satisfied with online consent to testing and suggested it was more convenient: “*Online, everybody prefers online”; “If it required me going somewhere to meet someone, then it probably would have taken me longer to get around to doing it”.* However, one patient referred to her age as the reason she would prefer in-person support: “*Well, I am over 70, I prefer to do things where I am speaking to someone”*.

A psychoeducational intervention (PEI) containing information about breast cancer genetic testing was explored by focus groups (paper version (50),) and semi-structured interviews (video format (61),). The paper PEI was visually attractive and culturally acceptable: “*I like the cover; you have a variety of ethnic groups and ages”* and appreciated as a take-home resource: “*you”re only going to remember a little piece of what [your health care professional says]…but hand me books…I can flip through it and then…write down notes to ask the next time I see somebody”; “I would see it; I can hold it; I can turn the pages…it prompts me to start thinking”* (50). Patients with breast cancer had emotional reactions to patient narratives in the video PEI: “*It just makes you realize [sic] that other people feel or felt like that….touching to watch the stories because I can relate, I kind of teared up”* (61).

Focus groups with 15 BRCA carriers with breast cancer guided development and evaluation of a web-based ptDA (63). Key decision-making motivating factors were identified, such as feeling obligated e.g., “*do the right thing”* to save life by having risk-reducing ovarian surgery, or HCP being strong influencers, e.g., “*my surgeon was gung-ho”,* describing consultations about risk-reducing mastectomies. Inclusion of a values-based exercise was appreciated: “*When it is in black and white in front of me and I am able to block out everybody else, what they want, what they think I should do and I can look at it and say what is the best thing step by step for me and then get a print out—that is huge”.* PtDA timing was debated, suggesting need for personalisation: “*The patient will probably let you know if they are ready for it or not”.* Patients preferred to use ptDAs in clinical settings: “*more geared up….more serious about it if it was in an office than at home”*, while clinicians (also included in focus groups) were keen for at-home use but cautioned that support was needed: “*…are they really going to know how to self-interpret with what they”ve just done?”*

## 4. Discussion

### 4.1. Discussion

This systematic literature review identified a range of decision support resources about genetic cancer susceptibility testing or cancer risk management for carriers. The heterogeneity of resources and study designs precluded meta-analysis. However, to meet study aims it was important that the systematic review search strategy was inclusive and broad to capture any types of resources, including digital, paper-based or educational that might benefit patients with different preferences and backgrounds. Any existing resources that could be delivered in current clinical practice or easily adapated might improve decision-making experience and outcomes, and needed to be considered.

Regarding the aims of this review:

i) decision support resources used to streamline cancer susceptibility genetic testing were non-inferior in terms of knowledge and decision satisfaction, however there was limited rigorous evaluation in randomised control trials compared to usual care. Decision support resources for carriers did not have a sustained effect on cancer-related distress, with transiently increased levels in one study possibly indicative of more deliberative decision-making. However, few studies included longer term follow-up beyond one to two months after resource use. Measures such as distress could change over time due to impact of the cancer diagnosis trajectory with changes in prognosis due to worsening disease. It is therefore important to make comparisons at multiple timepoints between patients with cancer who have used decision support resources to those who have not, to investigate the effect. Decisional conflict was low or moderate at baseline and with use of the intervention.
ii) all studies evaluating patient experience reported positive feedback on satisfaction and usefulness, however this was often by self-report, and many studies lacked a control group receiving usual care. There was a lack of patient diversity.

On balance, there was clear potential for decision support resources to be useful to help patients make more deliberative decisions in line with their personal values and situation, and may save time in clinic and healthcare resources. Taking into account the importance of varied patient preferences, there was no one best method of delivery, suggesting a flexible, multi-modal approach should be considered for future co-design of patient resources. This review highlighted several gaps in the availability of patient decision support resources to fulfil what patients in focus groups have told our research team that they want: trusted, up-to-date sources of information from experts that can also help them to educate their healthcare professionals and relatives (88).

Our findings align with results from a systematic review of Ottawa Decision Support Framework (ODSF)-based resources in 24 randomised controlled trials which showed PtDA used across a variety of medical specialities resulted in higher quality decisions and less HCP resource, compared to usual care (89). Some of the resources included in this review used a framework such as ODSF/IPDAS, but many did not which could have impacted effectiveness. The ODSF was recently updated following a review of use across 18 countries and >50,000 patients (90) to include decisional outcomes such as proportion of patients undecided, feeling uninformed, unsupported or unsure of values. There has been limited evaluation of these outcomes in decision support for genetic cancer susceptibility, and their inclusion should be given consideration in future research.

Streamlined, cost-effective pathways and patient resources are needed for HCP to deliver genomic testing “routinely to all people with cancer”, a commitment of the NHS Genomic Medicine Service (91) to inform surgical/treatment options, future cancer risks and risk to relatives. This is particularly relevant as genetic testing is moved into “mainstream” care, with patient-facing resources presenting an opportunity to more safely scale up delivery of testing without compromising informed decisions. Written or digital educational resources can be non-inferior to genetic counselling in the pre-test setting to increase knowledge (51, 54, 92).

Setting, mode of delivery and accessibility should be given due consideration; there was limited evaluation of this for the resources identified in this review. The results presented suggest people may not use a resource if asked to view a video/website/chatbot, or look at something at home, and they will rarely contact HCP with questions. It is not known whether these people did not need support or did not realise what support and benefit genetic counselling could provide. Those with more decision support needs should be referred to genetic counselling along with being offered tailored paper- and/or web-based patient resources because there is a suggestion that those with the greatest information needs may benefit most from additional decision support (43).

Quantifying genetic cancer susceptibility (known as “penetrance”) depends on the gene variant as well as age, medical and family history (93–96). Understanding of risk conferred by variants in cancer susceptibility genes has progressed at pace due to advances in genomic sequencing technology and large consortium studies (97–99). However, uncertainty remains about the likelihood that a carrier will develop cancer, which type of cancer and at what age. This is often experienced as trading one type of uncertainty for another, first finding out the genetic test result and then diverting thoughts and energy to thinking (or worrying) about what happens next. In patient-centred healthcare, uncertainty is multidimensional, including ethical (100), as well as scientific, system-based and personal factors linked to values, understanding and context (101, 102). Communication about uncertainty in a transparent, accessible way that engenders trust is a challenge for creators of ptDA, but if done successfully could improve understanding and emotional response (103–105) and make patients feel part of a team with their HCP (106). The findings of this review suggest the importance of including visual presentations of cancer risk to improve understanding of genetic cancer susceptibility and inform personalised decision-making.

Emerging research suggests that using digital technology such as smartphone applications could be acceptable and accessible for patients with lower literacy to consent for genetic testing (107), however continued bioethics exploration is needed to optimise inclusivitys. One size does not fit all, but harnessing digital technology to personalise decision support resources could empower more patients to take an active role in their care plan and improve health outcomes, consistent with the goals of the NHS Long Term Plan (108) and Universal Personalised Care Action Plan (109). This review has highlighted the usefulness, acceptability and time-saving nature of patient decision support resources, but has not identified one best method of delivery or any resources suitable for implementation in current clinical practice.

### 4.2. Future work

This review provides the groundwork to inform co-design with patients and other expert stakeholders from clinical genetics, oncology, charities, ethics, academic and health care bodies of a ptDA for Lynch syndrome, a genetic predisposition to certain cancers, mainly colorectal (bowel), endometrial (womb/uterine), ovarian and gastro-intestinal. The learning will be applied to create an adaptable template ptDA for genetic predispositions to other cancers. Most ptDA have focussed on pre-test decisions rather than genetic cancer susceptibility risk management. This highlighted the need for more resources for carriers, particularly for genes other than *BRCA1* and *BRCA2*. The ptDA we are co-designing is tailored based on personal characteristics to present risk estimates and relevant options spanning from targeted treatment of cancer to primary prevention of future cancers. This will be multi-modal to allow wider dissemination, using an interactive website with the option to print personalised paper-based versions, question checklists and summaries of values-based exercises to take to an appointment with a healthcare professional. The Person-Based Approach (110) is being used to develop and iteratively optimise content and delivery of the ptDA, with attention to accessibility such as for patients with lower health literacy.

Future research is needed in the following areas:

• to determine the best modes of implementation in clinical practice
• co-design of decision support resources with patients, including from diverse communities

### 4.3. Strengths and limitations

Non-peer reviewed, published resources may not have been identified. The decision to exclude pre-2011 publications resulted in loss of some relevant (but dated) evidence. Since genetics and technology are evolving so rapidly, it was decided that the review would be more relevant if it was focussed on more recent publications. However, even recent studies lacked exploration of the impact of more complex genetic testing, for example large gene panel tests or whole genome sequencing, which lead to more additional, unexpected findings and variants of uncertain significance requiring more interpretation and uncertainty about recommended management options.

The findings of this systematic review may be subject to uncertainty due to methodological limitations of the included studies, including: lack of comparison to usual care using randomised controlled trials; use of unvalidated and patient-reported outcome measures; potential bias due to missing data (included untracked usage of resources) and short-term follow-up. Samples often lacked carriers (where included, these were exclusively *BRCA1* and *BRCA2*). Studies were commonly conducted in a single centre or one country and therefore may not be generalisable.

### 4.4. Conclusions

More longitudinal research is needed regarding whether people complete actions in line with the decisions they make about cancer susceptibility genetic testing, cancer treatment and prevention. Longer term studies are important to understand whether people retain knowledge and accurate risk perception and maintain low levels of distress and decisional conflict over time after using decision support resources, compared to usual care. However, there has been little exploration of how measures such as distress might change over the longer term, and how this could be related to the psychological effects of a cancer diagnosis with changing prognosis, vs. the effect from using a PtDA which may be more transient. Evaluation should drive an iterative process of development and refinement of ptDA and determine the most effective mode of delivery in oncology and genetics services to improve health outcomes. Sustainable funding to update and securely host ptDA is essential to provide personalised cancer risk estimates and options based on current evidence and clinical guidelines.

Overall, the findings of this review suggest there is clear potential for ptDA and other decision support resources to complement the usual standard of care involving shared decision-making with healthcare professionals about genetic cancer susceptibility, but further development is needed to meet needs in current clincial practice. Psychological behavioural theory and a proven framework e.g., the ODSF (79) should underpin co-design, and quality standards should be met, e.g., IPDAS (24).

### 4.5. Practice implications

There are two main settings in which genetic cancer susceptibility patient decision support resources should be considered. First, healthcare professionals in oncology offering genetic cancer susceptibility testing for patients with cancer, where decisions to inform personalised treatment are often needed urgently. Patient-facing resources in this context could be brief and paper-based, with the option to delve into interactive, web-based resources and/or genetic counselling referral for those requiring or desiring a higher level of decision support. Secondly, post-genetic testing, carriers will need ongoing management of their lifelong condition, and ptDA can complement genetic counselling, encouraging decisions in accordance with personal values. Relatives of carriers will similarly need genetic counselling with a healthcare professional, but could additionally benefit from ptDA to increase knowledge pre-test.

A heterogeneous group of decision support resources has been identified in this review, each designed for a specific local care pathway and patient poulation. Clinical implementation shuld involves evaluating resources in complex care pathways, dealing with the realities of funding and staffing shortages present in the healthcare setting. Further research is needed to understand what decision support resources for genetic cancer susceptibility work for whom, how, why and in what setting (111, 112), with particular attention to patient values and preferences and ensuring inclusive accessibility.

## Data Availability

The original contributions presented in the study are included in the article/**Supplementary Material**, further inquiries can be directed to the corresponding author.
