# Supplementary material for: Patient decision support resources inform decisions about cancer susceptibility genetic testing and risk management: a systematic review of patient impact and experience
Source: Front Health Serv. 2023 May 31;3:1092816. doi: 10.3389/frhs.2023.1092816 (PMC10311450; doi:10.3389/frhs.2023.1092816)
Supplement: Supplementary file 1 [file Table1.docx]

**Supplementary Table 1.** MEDLINE search strategy

| **MEDLINE Search** | **Concept 1** | **Concept 2** | **Concept 3** |
| --- | --- | --- | --- |
| **Key concepts** | People with cancer, at risk of cancer, or family members considering genetic testing | Decision making | Written Intervention terms |
| **Free text terms / natural language terms**  (synonyms, UK/US terminology, medical/laymen’s terms, acronyms/abbreviations, drug brands, more narrow search terms)  *Consider: phrase searching, proximity operators, truncation, wildcards, field qualification (e.g. textword)* | (familial or genetic* or gene*1 or or heredit* or inherit* or predispos* or susceptib* or "famil* history*” or "TFGT" or "treatment focused genetic test*")  N2 (cancer* or neoplasm* or tumo?r* or malignan* or carcinoma* or sarcoma* or adenocarcinoma*) (53,765)  (“hereditary cancer*”  or “cancer predisposition”) (4,521)  Li Fraumeni or Li-Fraumeni or TP53 or BRCA1 or "BRCA 1" or BRCA2 or "BRCA 2" or PALB2 or "Breast Cancer 1" or "Breast Cancer 2" or Lynch or HNPCC or MLH1 or MSH2 or MSH6 or PMS2 or “Familial Adenomatous Polyposis” or FAP or APC (68,394) | Decision W1 (process* OR support* OR aid* OR tool* or making or -aid) (146,517)  “Choice behavi*”( 1,594)  “Patient preference” (3,933)  Decid* (88,647)  (“risk communicat*” or “risk assess*” or “risk inform*” or “risk apprais*” or “perceiv* risk” or “risk percep*”) n4 (tool* or method*) (7208) | (digital n3 (tool* or solution? or platform? or technolog* or dashboard? or portal) (6007)  ("database tool?" or "interactive tool?" or "software tool?" or "internet tool" or "internet intervention" or "electronic tool?" or "electronic device?" or "computer* assisted intervention*") (6009)  (digitiz* or digitis* or digitali*) (26400)  "patient portal?" (637)  ("e-technology" or "electronic technology" or "e-clinical”) (354)  "web portal?" (725)  (ipad or PDA or "personal digital assistant" or "mobile phone?" or "smart phone?" or smartphone? or "mobile app*" or "mobile technolog*" or "mobile health" or "mobile media" or "health app" or "m health" or palmtop? or laptop? or "hand held device?" or "text messag*" or SMS or IVR or "interactive voice recognition" or "voice activation" or "web deliv*") (46,856)  (interactive and website?) (1039)  ("web based" or "web tool?" or "web delivery" or "web delivered" or podcast*) (31,718)  ("e-mail" or email or "electronic mail*") (14184)  Handout? (1025)  Brochure? (2247)  Booklet? (3498)  Leaflet? (22004)  Intervention? (861,034)  Pamphlet? (1818)  Program* (854,368)  Material? (695,933)  Resource? (326,225)  Algorithm? (246,436)  Navigation? (23,118)  Aid? (163,159)  Framework (273,266)  Paper? (831,297) |
| **Controlled vocabulary** **terms / Subject terms**  (MeSH terms, Emtree terms)  *Consider: explode, major headings, subheadings* | Genes, BRCA1/ (3,533)  BRCA1 Protein/ (3,754)  Genes, BRCA2/ (2,175)  BRCA2 Protein/ (2,109)  Hereditary Breast and Ovarian Cancer Syndrome/ (220)  Lynch syndrome II (21)  EXP Genetic Testing/ and EXP neoplasms (9561)  Neoplastic syndromes, hereditary/ (2,090)  EXP genetic predisposition to disease/ and EXP neoplasms (36785)  EXP Disease susceptibility/ and MM neoplasms(40674)  = TOTAL:  (142,259 once neoplasms combined with each of the general disease susceptibility thesaurus terms to limit the results to cancer-related). | EXP Decision Making+ (200,616)  EXP Decision Support Techniques (76,873)  Risk reduction behaviour (5,023)    = TOTAL  (470,577) | Patient Navigation/ (571)  Patient Portals (303)  Patient Education Handout (415)  MM Mobile Applications/ (4568)  MM Communications Media/ (1140)  MM smartphone/ (3020)  EXP computer systems/ (177,776)  EXP internet/ (79,132)  MM Pamphlets/ (1782)  = TOTAL  (3,850,529) |
