# Supplementary material for: Patient decision support resources inform decisions about cancer susceptibility genetic testing and risk management: a systematic review of patient impact and experience
Source: Front Health Serv. 2023 May 31;3:1092816. doi: 10.3389/frhs.2023.1092816 (PMC10311450; doi:10.3389/frhs.2023.1092816)
Supplement: Supplementary file 2 [file Table2.docx]

**Supplementary Table 2.** List of conference proceedings and grey literature searches

|  | **Source and date(s)** | **n abstracts screened** | **Eligible references (not already found in database searches)** |
| --- | --- | --- | --- |
| 1 | Annual conference Clinical Genetics of Cancer  (identified by database searches) 2018 <https://hccpjournal.biomedcentral.com/articles/10.1186/s13053-018-0101-5> | 23 | 0 |
| 2 | Annual conference Clinical Genetics of Cancer  (identified by database searches) 2017 <https://www.ncbi.nlm.nih.gov/pmc/articles/PMC5841194/> | 21 | 0 |
| 3 | Annual conference of hereditary cancers 2016 <https://www.ncbi.nlm.nih.gov/pmc/articles/PMC5731602/> | 17 | 0 |
| 4 | Annual conference of hereditary cancers 2015 <https://paperity.org/p/84782121/meeting-abstracts-from-the-annual-conference-on-hereditary-cancers-2015> | 13 | 0 |
| 5 | 1^st^ International symposium on Hereditary Breast and Ovarian Cancer: 2005 | Not found |  |
| 6 | 2^nd^ International symposium on Hereditary Breast and Ovarian Cancer: 2007 | Not found |  |
| 7 | 3^rd^ International symposium on Hereditary Breast and Ovarian Cancer: 2010 <https://www.ncbi.nlm.nih.gov/pmc/articles/PMC2768503/> | 73 | 0 |
| 8 | 4^th^ International symposium on Hereditary Breast and Ovarian Cancer: 2012 <https://www.ncbi.nlm.nih.gov/pmc/articles/PMC3320236/> | 100 | 0 |
| 9 | 5^th^ International symposium on Hereditary Breast and Ovarian Cancer: 2014 <https://europepmc.org/article/pmc/pmc3997469> | 137 | 1 |
| 10 | 6^th^ International symposium on Hereditary Breast and Ovarian Cancer 2016 <https://www.ncbi.nlm.nih.gov/pmc/articles/PMC4900850/> | 154 | 0 |
| 11 | 7^th^ International symposium on Hereditary Breast and Ovarian Cancer 2018 <https://www.ncbi.nlm.nih.gov/pmc/articles/PMC6023566/> | 163 | 2 |
| 12 | NHS Evidence  hereditary cancer decision aid Note: unable to use quotations, truncation or adjacency <https://www.evidence.nhs.uk/> [accessed 03/07/2020] | 197 | 0 |
| 13 | TRIP (Turning Research Into Practice)  <https://www.tripdatabase.com/> [accessed 03/07/2020] (title:(hereditary or familial or predisposition or susceptibility) and cancer)(title:Decision (process* OR support* OR aid* OR tool* or making or aid) )(title:(handout* or brochure* or booklet* or leaflet* or paper or intervention* or web* or digital* or online or internet or pamphlet* or program* or material* or resource* or algorithm* or tool or navigation* or technolog* or aid*) ) | 8 | 0 |
| 14 | Google Scholar <https://scholar.google.com/> [accessed 27/11/2020] All words: gene*, decision* At least one of: cancer, BRCA* Location: anywhere in article | First 200 of 29300 | 1 |
| 15 | Google Scholar [accessed 27/11/2020] All words: heredit*, cancer Exact phrase: genetic testing At least one of: intervention or aid or decision Location: anywhere in article | First 200 of 18500 | 0 |
| 16 | Google Scholar [accessed 27/11/2020] All words: cancer, decision* Exact phrase: genetic testing Location: anywhere in article | First 200 of 78100 | 0 |
