# Supplementary material for: Patient decision support resources inform decisions about cancer susceptibility genetic testing and risk management: a systematic review of patient impact and experience
Source: Front Health Serv. 2023 May 31;3:1092816. doi: 10.3389/frhs.2023.1092816 (PMC10311450; doi:10.3389/frhs.2023.1092816)
Supplement: Supplementary file 3 [file Table3.docx]

**Supplementary Table 3.** Critical appraisal

For quantitative studies, the National Institute for Health and Care Excellence (NICE) quantitative intervention studies checklist was used [37]. Aspects of study design and reporting were appraised. Each study was then awarded an overall study quality grading for internal validity and external validity (see legend). For qualitative studies, the NICE quality appraisal checklist for qualitative studies was used [37]. Aspects of study design and reporting were appraised. Each study was then subject to an overall assessment grading of how well the study was conducted, as far as could be ascertained from the paper (see legend). For mixed-methods studies, the mixed methods appraisal tool (MMAT) was used [38]. Responses to appraisal of study criteria in the table are presented as yes, no, or N/A (not applicable or not able to ascertain from the paper).

| **MMAT Quant 3.3** | **MMAT Quant 3.4** | **MMAT Quant3.5** |
| --- | --- | --- |
| yes | N/A | no |
| yes | no | no |

**Legend:**
++ All or most of the checklist criteria have been fulfilled, where they have not been fulfilled the conclusions are very unlikely to alter.

+ Some of the checklist criteria have been fulfilled, where they have not been fulfilled, or not adequately described, the conclusions are unlikely to alter.

− Few or no checklist criteria have been fulfilled and the conclusions are likely or very likely to alter.
